# *In vitro* Susceptibility to β-Lactam Antibiotics and Viability of *Neisseria gonorrhoeae* Strains Producing Plasmid-Mediated Broad- and Extended-Spectrum β-Lactamases

**DOI:** 10.3389/fmicb.2022.896607

**Published:** 2022-06-20

**Authors:** Ilya Kandinov, Dmitry Gryadunov, Alexandra Vinokurova, Olga Antonova, Alexey Kubanov, Victoria Solomka, Julia Shagabieva, Dmitry Deryabin, Boris Shaskolskiy

**Affiliations:** ^1^Center for Precision Genome Editing and Genetic Technologies for Biomedicine, Engelhardt Institute of Molecular Biology, Russian Academy of Sciences, Moscow, Russia; ^2^State Research Center of Dermatovenerology and Cosmetology, Russian Ministry of Health, Moscow, Russia

**Keywords:** *Neisseria gonorrhoeae*, β-lactamase-producing plasmids, *N. gonorrhoeae* viability, extended-spectrum β-lactamase, antimicrobal susceptibility

## Abstract

*Neisseria gonorrhoeae* plasmids can mediate high-level antimicrobial resistance. The emergence of clinical isolates producing plasmid β-lactamases that can hydrolyze cephalosporins, the mainstay treatment for gonorrhea, may be a serious threat. In this work, *N. gonorrhoeae* strains producing plasmid-mediated broad- and extended-spectrum β-lactamases (ESBLs) were obtained *in vitro*, and their viability and β-lactam antibiotic susceptibility were studied. Artificial p*bla*_TEM-1_ and p*bla*_TEM-20_ plasmids were constructed by site-directed mutagenesis from a p*bla*_TEM-135_ plasmid isolated from a clinical isolate. Minimum inhibitory concentration (MIC) values for a series of β-lactam antibiotics, including benzylpenicillin, ampicillin, cefuroxime, ceftriaxone, cefixime, cefotaxime, cefepime, meropenem, imipenem, and doripenem, were determined. The *N. gonorrhoeae* strain carrying the p*bla*_TEM-20_ plasmid exhibited a high level of resistance to penicillins and second–fourth-generation cephalosporins (MIC ≥2 mg/L) but not to carbapenems (MIC ≤0.008 mg/L). However, this strain stopped growing after 6 h of culture. The reduction in viability was not associated with loss of the plasmid but can be explained by the presence of the plasmid itself, which requires additional reproduction costs, and to the expression of ESBLs, which can affect the structure of the peptidoglycan layer in the cell membrane. Cell growth was mathematically modeled using the generalized Verhulst equation, and the reduced viability of the plasmid-carrying strains compared to the non-plasmid-carrying strains was confirmed. The cell death kinetics of *N. gonorrhoeae* strains without the p*bla*_TEM-20_ plasmid in the presence of ceftriaxone can be described by a modified Chick–Watson law. The corresponding kinetics of the *N. gonorrhoeae* strain carrying the p*bla*_TEM-20_ plasmid reflected several processes: the hydrolysis of ceftriaxone by the TEM-20 β-lactamase and the growth and gradual death of cells. The demonstrated reduction in the viability of *N. gonorrhoeae* strains carrying the p*bla*_TEM-20_ plasmid probably explains the absence of clinical isolates of ESBL-producing *N. gonorrhoeae*.

## Introduction

The development of multidrug resistance in the pathogen *Neisseria gonorrhoeae* is a major problem worldwide. According to the World Health Organization (WHO), gonorrhea may become incurable due to the ineffectiveness of old antimicrobials and the lack of new drugs for its treatment (Tacconelli et al., 2018; Golparian and Unemo, 2022).

Currently, and possibly in future, antibiotics of the β-lactam group are the preferred drugs for treating gonococcal infection (Workowski and Bolan, 2015; WHO, 2016; Tacconelli et al., 2018; Unemo et al., 2020). This group of antibiotics includes the penicillin, cephalosporin, carbapenem, and monobactam classes. The mode of action of β-lactam antibiotics is to disrupt the synthesis of the peptidoglycan layer in bacterial cell walls, leading to bacterial cell death. In the mid-1940s, the discovery of the penicillins started a revolution in the treatment of gonococcal infection, and penicillins became the gold standard of treatment, resulting in cure after a single injection. However, by the end of the 20th century, most clinical isolates of *N. gonorrhoeae* showed reduced susceptibility or resistance to penicillins; thus, the use of penicillins was discontinued (Unemo and Shafer, 2014; Unemo and Jensen, 2017).

Currently, the preferred drugs for the treatment of gonococcal infections include another class of β-lactams—third-generation cephalosporins, i.e., ceftriaxone, cefotaxime and cefixime, which are used alone or in combination with the macrolide antibiotic azithromycin (Workowski and Bolan, 2015; WHO, 2016; Tacconelli et al., 2018; Unemo et al., 2020). However, the levels of gonococcal resistance to cephalosporins are increasing annually, and treatment failure with these drugs has already been reported in several countries (Unemo et al., 2012; Allen et al., 2013; Golparian et al., 2014; Attram et al., 2019). The next class of β-lactams that could replace cephalosporins if cephalosporin resistance spreads is the carbapenems (Unemo et al., 2020).

The resistance of *N. gonorrhoeae* to β-lactam antibiotics is associated with both chromosomal and plasmid determinants. Chromosomal mutations include substitutions in penicillin-binding proteins 1 and 2 (PBP1 and PBP2), mutations in the porin protein PorB that lead to a change in cell membrane permeability, and mutations that cause an increase in the expression level of the MtrCDE efflux pump (Unemo and Shafer, 2014; Unemo and Jensen, 2017; Unemo et al., 2019). In this resistance mechanism, the presence of the β-lactamase enzyme, which hydrolyzes the C-N bond in the β-lactam ring of the antibiotic and inactivates the drug, is particularly important. The proportion of *N. gonorrhoeae* clinical isolates carrying the p*bla*_TEM_ plasmid is not very high; for example, in the Russian population, the percentage remains at ~5% of the total number of isolates (Kubanov et al., 2019). However, the presence of β-lactamases in gonococci causes a significant increase in the level of resistance to penicillins (MIC_pen_ ≥ 16 mg/L) compared to that related to chromosomal mutations (MIC_pen_ = 0.12–1.0 mg/L; Shaskolskiy et al., 2019; Młynarczyk-Bonikowska et al., 2020). To date, clinical isolates of *N. gonorrhoeae* have been identified to produce only enzyme variants that are broad-spectrum β-lactamases (penicillinases), which cannot hydrolyze cephalosporins or carbapenems.

The following types of p*bla*_TEM_ plasmids have been identified in gonococci: Asian (7,426 bp), African (5,599 bp), Toronto/Rio (5,154 bp), Nimes (6,798 bp), New Zealand (9,309 bp), Johannesburg (4,865 bp) and Australian (3,269 bp; Müller et al., 2011). The plasmid-encoded *bla* gene has a length of 861 bp. Most penicillinase-producing isolates of *N. gonorrhoeae* carry the plasmid containing the *bla*_TEM-1_ gene, but recently, the *bla*_TEM-135_ variant has been increasingly detected in the worldwide population of *N. gonorrhoeae* (Ohnishi et al., 2010; Nakayama et al., 2012; Muhammad et al., 2014; Cole et al., 2015; Tanaka et al., 2021). The *bla*_TEM-1_ gene has been found in both African and Asian plasmids, while *bla*_TEM-135_ is present mainly in Toronto/Rio and Asian plasmids (Cole et al., 2015; Yan et al., 2019). *bla*_TEM-135_ differs from *bla*_TEM-1_ in a single-nucleotide thymine-to-cytosine substitution at codon 182 (ATG → ACG). The resulting Met182Thr mutation is located far from the enzyme active site (17 Å) in the hinge region between two β-lactamase domains and leads to stabilization of the β-lactamase structure (Orencia et al., 2001). As previously noted (Cole et al., 2015; Yan et al., 2019), the increased enzyme stability may have contributed to the persistence of the TEM-135 β-lactamase variant in the *N. gonorrhoeae* population.

A large group of enzymes are related to bacterial β-lactamases. TEM-1 and TEM-135 are class A serine β-lactamases according to the Ambler classification (Bradford, 2001; Bush and Jacoby, 2010; Tooke et al., 2019). This class also includes extended-spectrum β-lactamases (ESBLs), such as TEM-20, which can hydrolyze both penicillins and cephalosporins. The *bla* gene variant encoding the TEM-20 β-lactamase can be produced by just a single-nucleotide substitution (GGT → AGT) in the *bla*_TEM-135_ gene, which results in the Gly238Ser mutation (Arlet et al., 1999; Bradford, 2001). This substitution is associated with a change in the β-lactamase conformation, which increases the flexibility of the enzyme active site; as a result, the β-lactamase gains the ability to bind cephalosporins without losing its penicillin-hydrolyzing character (Orencia et al., 2001; Yan et al., 2019). Although *N. gonorrhoeae* clinical isolates carrying the p*bla*_TEM-20_ plasmid have not been found in nature, just a single change could allow gonococci to encode an ESBL, which could end the therapeutic use of third-generation cephalosporins.

The goals of this work were to construct p*bla*_TEM_ plasmids that contain different variants of the *bla* gene, to produce genetically engineered *N. gonorrhoeae* strains that contain p*bla*_TEM_ plasmids, to study the properties of *N. gonorrhoeae* strains transformed with these plasmids and to assess the viability and β-lactam antibiotic (penicillins, cephalosporins and carbapenems) resistance of these strains. Particular attention was placed on constructing the p*bla*_TEM-20_ plasmid and studying the properties of the *N. gonorrhoeae* strain carrying this plasmid. Such a strain may express the TEM-20 β-lactamase, which can hydrolyze both penicillins and cephalosporins. Studies on laboratory mutants generated by *in vitro* mutagenesis contribute to our understanding of evolutionary pathways and enable predictions of future events. This research will allow the assessment of risks associated with the emergence and spread of *N. gonorrhoeae* clinical isolates with resistance to third-generation cephalosporins and the possibility to further use β-lactam antibiotics to treat gonococcal infections.

## Materials and Methods

All procedures and experiments with *N. gonorrhoeae*, including transforming cells, isolating plasmid DNA, electroporating cells, culturing cells on solid media, washing cells in Petri dishes and passaging them to other dishes, counting the colonies formed and assessing the number of viable bacteria (i.e., colony-forming units, CFU), were performed as described previously (Spence et al., 2008; Dillard, 2011).

All procedures with *N. gonorrhoeae* strains including transformation, estimation of susceptibility to antimicrobials and assessment of cell viability were conducted in biological safety cabinet (BSC) of the State Research Center of Dermatovenerology and Cosmetology, Russian Ministry of Health. The BSC was equipped with SafeFAST Elite class III microbiological safety cabinet (Faster, Italy). The laboratory room and equipment were carefully sterilized after each experiment using AntiseptiX decontamination reagents (Biomedical Innovations, Russia) and a UVR-Mi bactericidal air recirculator (Biosan, Latvia). Sterilization of culture vessels and disposable microbiological accessories was performed twice after each experiment at 130°C for 30 min using an autoclave (Tuttnauer, United States).

All strains were killed by heat inactivation after the experiments. Petri dishes with cells were autoclaved at 130°C for 30 min before disposing.

### Isolation of the p*bla*_TEM-135_ Plasmid From a Clinical Strain of *Neisseria gonorrhoeae* With Natural Resistance to Penicillins

We used an *N. gonorrhoeae* clinical isolate from the collection of the State Scientific Center of Dermatovenerology and Cosmetology of the Ministry of Health of the Russian Federation that was obtained in 2017 from the Chuvash Republic; this isolate carried a Toronto/Rio p*bla*_TEM-135_ plasmid, and the associated minimum inhibitory concentration of penicillin (MIC_pen_) was ≥32 mg/L (Shaskolskiy et al., 2019). The clinical isolate was seeded on GC chocolate agar (Thermo Fisher Scientific, United States) supplemented with IsoVitaleX Enrichment (Becton-Dickinson, United States) and 16 mg/L benzylpenicillin (Sigma–Aldrich, United States). The dish was incubated overnight at 37°C in 5% CO_2_. Formed colonies were harvested using a culture loop in 100 μl of phosphate-buffered saline (PBS). Plasmid DNA was isolated with a Monarch Plasmid Miniprep Kit/T1010 (New England Biolabs, United Kingdom). The concentration and purity of the DNA preparations were determined using a Nanodrop 2000 spectrophotometer (Thermo Fisher Scientific, United States). Plasmid DNA was analyzed by PCR as described previously (Palmer et al., 2000; Shaskolskiy et al., 2019).

### Construction of Plasmids With Different Variants of the *bla* Gene on the Basis of p*bla*_TEM-135_ Isolated From a Clinical *Neisseria gonorrhoeae* Strain

**Figure 1:**
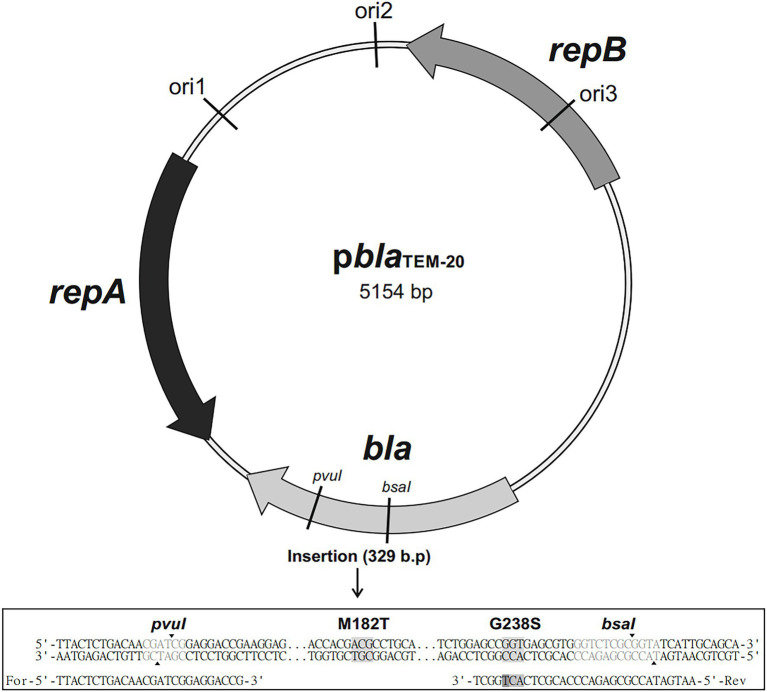
Map of the p*bla*_TEM-20_ plasmid (5,154 base pairs long) containing the *bla*_TEM-20_ gene associated with resistance to β-lactam antibiotics, including penicillins and cephalosporins. The plasmid contained three replication origins (ori1, ori2, and ori3) compatible with Gram-negative bacteria and the *repA* and *repB* genes encoding replication initiation proteins. The nucleotide sequence alignment of a 329 bp insert that contained the portion of the *bla* gene with the GGT → AGT mutation, which resulted in the Gly → Ser substitution at codon 238, and the ATG → ACG mutation, which resulted in the Met→Thr substitution at codon 182, is shown below.

The *bla* gene fragment was amplified by simultaneous site-directed mutagenesis, which ensured that the mutations were introduced into the gene. The primers 5′-TTACTTCTGACAACGATCGGAGGACCGAAGG-3′-FOR and 5′-AATGATACCGCGAGAC CCACGCTCACTGGCT-3′-REV were used to obtain a PCR-amplified fragment of the *bla* gene that harbored the GGT → AGT mutation that results in the Gly238Ser substitution and is present in the TEM-20 ESBL (Figure 1). A PCR fragment corresponding to the sequence of the *bla* gene that encodes the TEM-1 broad-spectrum β-lactamase was produced by a similar method.

The PCR fragments and the p*bla*_TEM-135_ plasmid were digested with the restriction endonucleases PvuI and BsaI. Then, the linearized p*bla*_TEM-135_ plasmid and the PCR fragments with sticky ends were treated with a ligation mixture to generate the final p*bla*_TEM-1_ and p*bla*_TEM-20_ plasmids. The map of the resulting p*bla*_TEM-20_ plasmid is shown in Figure 1.

### Transformation of *Escherichia coli* Cells

The ready-to-use 10-beta competent *E. coli* strain DH10B (New England Biolabs, United Kingdom) was used for plasmid transformation. The puc19 (K+) vector was used as the transformation control, and a sample without DNA was used as the negative control. Cells were grown and subsequently reseeded on selective media. Medium containing 256–512 mg/L benzylpenicillin was used for transformation of *E. coli* with the p*bla*_TEM-135_ and p*bla*_TEM-1_ plasmids; medium containing 64 mg/L ceftriaxone (Sigma–Aldrich, United States) was used for transformation with the p*bla*_TEM-20_ plasmid.

*E. coli* colonies formed on the selective media were collected with a culture loop in PBS, and plasmid DNA was isolated using a Plasmid Miniprep/BC021S Kit (Evrogen, Russia). The *bla* gene sequence and p*bla*_TEM_ plasmid type were confirmed by PCR and Sanger sequencing.

### Transformation of *Neisseria gonorrhoeae* Cells

We used *N. gonorrhoeae* strain ATCC 49226 (F-18, CDC 10, 001, P935), a reference strain for antimicrobial susceptibility testing.^1^ *Neisseria gonorrhoeae* were transformed with the *pbla*_TEM-1_, p*bla*_TEM-135_ and p*bla*_TEM-20_ plasmids, which were purified from *E. coli* cells, by electroporation.

The optimal condition for transformation was as follows. *Neisseria gonorrhoeae* cells were collected from the overnight culture inoculum with a sterile culture loop and resuspended in 0.3 M sucrose. The cells were precipitated at 12,000 rpm and resuspended in 0.3 M sucrose, and this procedure was repeated 2–3 times. For electroporation, 100–200 μl of the purified cell suspension and 50–100 ng of plasmid DNA were put into 2 mm electroporation cuvettes. A suspension of cells without the addition of plasmid DNA was also subjected to electroporation and used as the “wild-type” strain in subsequent experiments. Electroporation was performed by applying single pulsed discharges of 2.5 kV, 200 Ω, and 25 μF. The cooled cell suspensions were plated on chocolate agar dishes, washed and subcultured on dishes with a selective antibiotic: for strains carrying the p*bla*_TEM-1_ and p*bla*_TEM-135_ plasmids, 2 mg/L penicillin was used, and for strains carrying the p*bla*_TEM-20_ plasmid, 0.5 mg/L ceftriaxone was used.

The wild-type *N. gonorrhoeae* strain and the strains carrying the p*bla*_TEM-1_ and p*bla*_TEM-135_ plasmids were stored at −80°C in cryopreservation medium containing trypticase-soy broth and glycerol at a ratio of 4:1.

The expression of β-lactamases in transformed cells was checked using nitrocefin disks (Remel, United States), which qualitatively detect the presence of enzymes, including penicillinases and cephalosporinases, that are capable of destroying the β-lactam ring in the substrate (nitrocefin).

### Estimation of the Susceptibility of *Neisseria gonorrhoeae* to β-Lactam Antibiotics

The following β-lactam antibiotics were used in this work: benzylpenicillin; ampicillin (extended-spectrum aminopenicillin); cefuroxime (second-generation cephalosporin); ceftriaxone, cefixime and cefotaxime (third-generation cephalosporins); cefepime (fourth-generation cephalosporin); and meropenem, imipenem, and doripenem (carbapenems). All antibiotics were obtained from Sigma–Aldrich (United States).

The MICs of antibiotics for *N. gonorrhoeae* strains were measured by a serial dilution method. *Neisseria gonorrhoeae* cells were cultured on GC chocolate agar supplemented with IsoVitaleX Enrichment. Inocula (0.5 McFarland) of the wild-type *N. gonorrhoeae* strain (ATCC 49226) and the strains carrying the p*bla*_TEM_ plasmids were prepared. Microorganisms were seeded in Petri dishes containing selective medium supplemented with different concentrations of antibiotics. After 24 h of incubation at 37°C in 5% CO_2_, the presence/absence of bacterial growth in the Petri dishes was assessed. Based on the measurement results, each strain was characterized in accordance with the established criteria of the European Committee on Antimicrobial Susceptibility Testing (EUCAST): S–susceptible, R–resistant.^2^

### Assessment of *Neisseria gonorrhoeae* Cell Viability: Cell Growth and Death Studies

To estimate the effects of the plasmids on the survival of gonococci in the presence of antibiotics, the viability of *N. gonorrhoeae* cells carrying p*bla*_TEM_ plasmids was studied by constructing growth curves for cells cultured in the absence of antibiotics and death curves for cells cultured in the presence of ceftriaxone. The change in the number of viable cells (i.e., CFU) over time was determined by counting the number of colonies formed on solid medium.

All experiments conducted to study *N. gonorrhoeae* cell growth and death were performed immediately after transformation, allowing us to avoid cell storage. The avoidance of storage was important because of the low viability of *N. gonorrhoeae* cells carrying the p*bla*_TEM-20_ plasmid.

To study cell growth, Petri dishes containing chocolate agar (10 ml) without the addition of antibiotics were used. An inoculum of cells (0.5 McFarland) was diluted to ~50 CFU/ml, and 1 ml of the cell suspension was plated in each dish, yielding nine dishes containing ~50 cells/dish for each *N. gonorrhoeae* strain. Every hour, the cultured cells were removed from every dish by washing as follows: 1 ml of a sterile 0.3 M sucrose solution was poured onto the agar surface, the cells were resuspended in the dish with a sterile plastic loop without disturbing the agar surface layer, and the resulting cell suspension was transferred to a new dish. Thus, the cells were cultured in dish No. 1 for 1 h, in dish No. 2 for 2 h, and so on. After the cultured cells were removed by washing, the dishes containing the removed cells were incubated for 24–48 h at 37°C in 5% CO_2_, and the colonies formed were counted.

To study cell death in the presence of ceftriaxone, an inoculum of cells (0.5 McFarland) was diluted to ~1,000 CFU/ml, and 1 ml of the cell suspension was plated in each dish supplemented with ceftriaxone, yielding nine dishes containing ~1,000 cells/dish for each *N. gonorrhoeae* strain. Ceftriaxone concentrations of 0.03, 0.125, and 2 mg/L were used. Every hour, the cultured cells were removed from every dish by washing, and the resulting cell suspensions were transferred to new dishes without the addition of ceftriaxone. The dishes were incubated as described above, and the colonies formed were counted. All experiments conducted to study the growth and death of the obtained clones were performed in triplicate.

### Mathematical Models of Cell Growth and Death

The generalized Verhulst equation was used to model cell growth for all strains under study (Peleg et al., 2007; Peleg and Corradini, 2011). To construct death curves for the strains not carrying the p*bla*_TEM-20_ plasmid cultured in the presence of ceftriaxone, a modified Chick–Watson model (Jensen, 2010; Peleg, 2021) was used. The parameters were fit to the equation by determining the numerical solutions of the Cauchy problem using MATLAB 2021b software for different *r*, *N_asymp_*, and α values for the Verhulst equation and for different *k_obs_* values for the Chick–Watson law. Optimization of the obtained numerical solutions compared with the experimental results was carried out by the least squares method. The statistical significance of the difference between the mathematical models, which describe experimental results for different data series, was evaluated by Fisher’s F test (F test) in accordance with a procedure described previously (Motulsky and Ransnas, 1987).

## Results

### Production of the p*bla*_TEM-1_, p*bla*_TEM-135_, and p*bla*_TEM-20_ Constructs

The p*bla*_TEM-135_ plasmid obtained from the clinical *N. gonorrhoeae* isolate was used to construct the artificial p*bla*_TEM-1_ and p*bla*_TEM-20_ plasmids. Three 5,154 bp plasmids were constructed; these plasmids differed in the *bla* gene structure (Figure 1) as follows:

p*bla*_TEM-1_, no mutations in codons 182 and 238;

p*bla*_TEM-135_, the ATG → ACG substitution at codon 182 leading to the Met182Thr substitution, no mutation at codon 238; and

p*bla*_TEM-20_, ATG → ACG substitution at codon 182 leading to the Met182Thr substitution and the GGT → AGT substitution at codon 238 leading to the Gly238Ser substitution.

These plasmids could replicate in both *E. coli* and *N. gonorrhoeae* cells due to the presence of replication origins compatible with gram-negative bacteria and replication initiation proteins.

### Production of *Escherichia coli* and *Neisseria gonorrhoeae* Strains Carrying p*bla*_TEM_ Plasmids

*E. coli* and *N. gonorrhoeae* strains carrying the p*bla*_TEM-1_, p*bla*_TEM-135_, and p*bla*_TEM-20_ plasmids were generated. *E. coli* strains carrying the p*bla*_TEM_ plasmids were grown on selective media with antibiotics and stored in Petri dishes at 4°C.

The *N. gonorrhoeae* strains carrying the p*bla*_TEM-1_ and p*bla*_TEM-135_ plasmids retained their viability after several (4–5) passages from dishes and after storage in cryopreservation medium. However, the *N. gonorrhoeae* strain carrying the p*bla*_TEM-20_ plasmid could not be stored in cryopreservation medium. In addition, cell viability was lost after 6 h of incubation on plates; colonies did not grow after subculture on medium with or without antibiotics (penicillin and ceftriaxone). The loss of viability cannot be explained by the loss of plasmids, since the test on nitrocefin disks produced positive results, indicating the presence of β-lactamases. Moreover, the presence of p*bla*_TEM_ plasmids with the introduced mutations in the β-lactamase gene was confirmed by both PCR and Sanger sequencing.

### Antimicrobial Susceptibility Testing of *Neisseria gonorrhoeae* Strains Carrying Different p*bla*_TEM_ Plasmids

The susceptibility of *N. gonorrhoeae* strains carrying various p*bla*_TEM_ plasmids to several groups of β-lactam antibiotics was studied. The antibiotics included penicillins, which were previously used to treat gonococcal infections; cephalosporins, which are currently used for gonorrhea treatment; and carbapenems, which may replace cephalosporins if cephalosporin resistance spreads (Unemo et al., 2020). The MICs measured for these drugs are shown in Table 1.

**Table 1:** Susceptibility of *Neisseria gonorrhoeae* strains carrying different p*bla*_TEM_ plasmids to β-lactam antibiotics.

Antibiotic	EUCAST criteria	MIC, mg/L			
		WT^*^	p*bla*_TEM-1_	p*bla*_TEM-135_	p*bla*_TEM-20_
Benzylpenicillin (PEN)	S: MIC_pen_ ≤ 0.06 R: MIC_pen_ > 1	0.25	16 (R)	32 (R)	16 (R)
Ampicillin (AMP)	–	0.125	8	8	16
Cefuroxime (CXM)	–	0.008	0.004	0.004	2
Ceftriaxone (CRO)	S: MIC_cro_ ≤ 0.125 R: MIC_cro_ > 0.125	0.03 (S)	0.015 (S)	0.03 (S)	4 (R)
Cefixime (CFM)	S: MIC_cfm_ ≤ 0.125 R: MIC_cfm_ > 0.125	0.015 (S)	0.015 (S)	0.015 (S)	16 (R)
Cefotaxime (CTX)	S: MIC_ctx_ ≤ 0.125 R: MIC_ctx_ > 0.125	0.015 (S)	0.008 (S)	0.015 (S)	8 (R)
Cefepime (FEP)	–	0.03	0.015	0.015	16
Meropenem (MEM)	–	0.002	0.002	0.002	0.004
Imipenem (IPM)	–	0.004	0.008	0.004	0.008
Doripenem (DOR)	–	0.002	0.002	0.002	0.002

S–susceptible and R–resistant according to the EUCAST criteria (EUCAST, 2022).

* WT–wild-type *N. gonorrhoeae*, ATCC 49226, not carrying the pbla_TEM_ plasmid.

After transformation of the wild-type *N. gonorrhoeae* strain with the p*bla*_TEM-1_ and p*bla*_TEM-135_ plasmids, which contain genes encoding a broad-spectrum β-lactamase (penicillinase), the strain exhibited a sharp decrease in susceptibility to antibiotics from the penicillin class (MIC_pen_ = 16–32 mg/L, MIC_amp_ = 8 mg/L). The MICs measured on solid media confirmed that the TEM-1 and TEM-135 enzymes were unable to hydrolyze cephalosporins and carbapenems.

When incubated for 24 h on plates with antibiotics, the *N. gonorrhoeae* strain carrying the p*bla*_TEM-20_ plasmid exhibited visible growth, allowing us to determine the MICs of β-lactams (Table 1). Even if this strain stopped growing after 6 h of culture, we could record the numbers of colonies on the plates after 24 h and estimate the MICs of the antibiotics. The MICs for the strain containing the p*bla*_TEM-20_ plasmid confirmed that the expressed TEM-20 β-lactamase is an ESBL that can hydrolyze both penicillins and cephalosporins of different generations. The cephalosporin MICs were above the EUCAST breakpoint for susceptibility/resistance, which was established to be 0.125 mg/L (Table 1). For example, the MICs of the third- and fourth-generation cephalosporins were at least 4 mg/L. Thus, the MIC of the antibiotic for the strain carrying the p*bla*_TEM-20_ plasmid exceeded the breakpoint established for third- and fourth-generation cephalosporins by more than a fivefold dilution.

The measured MIC of carbapenems did not exceed 0.008 mg/L for any *N. gonorrhoeae* strain, i.e., all strains were susceptible to carbapenems, proving that the TEM-1, TEM-135, and TEM-20 β-lactamase variants cannot hydrolyze carbapenems (EUCAST breakpoints for carbapenems are not available).

### Growth Curves of *Neisseria gonorrhoeae* Strains Carrying Different p*bla*_TEM_ Plasmids and the Mathematical Model of Cell Growth

**Figure 2:**
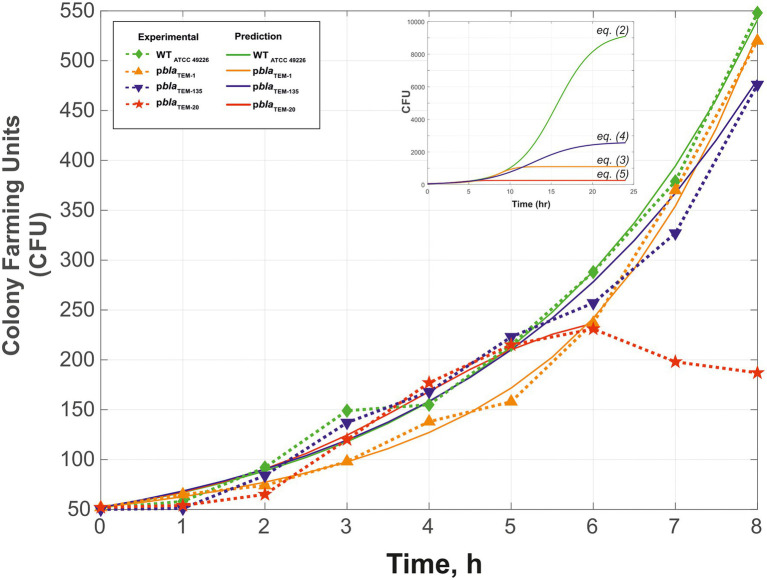
Growth curves (change in CFU over time) of *Neisseria gonorrhoeae* strain ATCC 49226 not carrying plasmids (WT) and carrying p*bla*_TEM_ plasmids, in the absence of antimicrobials (37°C, 5% CO_2_). The experimental results are indicated by the dashed lines and dots, and the theoretical curves constructed using Eqs. (2)–(5) are indicated by the solid lines. The inset shows the theoretical curves for incubation periods of up to 24 h to reach *N_asymp_*. In the graphs, the dots correspond to the mean CFU from three independent experiments (the initial CFU and the standard deviations are provided in Supplementary Table S1).

Figure 2 shows the growth curves of the *N. gonorrhoeae* cells carrying the p*bla*_TEM-1_, p*bla*_TEM-135_, and p*bla*_TEM-20_ plasmids compared to the growth curve of the wild-type cells in the absence of antibiotics. Notably, in our experiments, we determined the number of viable cells by determining the number of colonies formed (i.e., CFU) in a dish. The wild-type strain and the strains carrying the p*bla*_TEM-1_ and p*bla*_TEM-135_ plasmids exhibited growth throughout the 8 h of culture. However, the number of viable *N. gonorrhoeae* cells carrying the p*bla*_TEM-20_ plasmid began to decrease after 6 h of culture.

To compare the *N. gonorrhoeae* strains carrying different plasmids, we performed mathematical modeling of the cell growth kinetics. The generalized Verhulst equation [Peleg et al., 2007; Peleg and Corradini, 2011; Eq. (1)] was selected for modeling since it is the simplest and most universal equation for describing population growth dynamics until the transition to stationary phase.


(1) $$\frac{dN\left(t\right)}{dt}=rN{\left(t\right)}^{\alpha }\left(1-\frac{N\left(t\right)}{N_{asymp}}\right)$$

where *dN*(*t*)*/dt* is the growth rate at a given time, *r* is the proportionality coefficient, *N*(*t*) is the number of cells at a given time, *N_asymp_* is the maximum possible number of cells of a given type that can grow under the given experimental conditions, and α is the constant characterizing the growth specificity (*α* < 1 means that the organism does not achieve its potential for exponential growth, and *α* > 1 means that the organism exceeds this potential; Peleg et al., 2007).

Fitting of the parameters to Eq. (1) by determining the numerical solutions of the Cauchy problem for different *r*, *N_asymp_*, and α values resulted in Eqs. (2)–(5), which describe the growth curves of cells carrying and not carrying plasmids (Figure 2).

*N. gonorrhoeae* cells not carrying plasmids (wild-type):


(2) $$\frac{dN\left(t\right)}{dt}=0.188\;N{\left(t\right)}^{1.093}\left(1-\frac{N\left(t\right)}{9300}\right)$$

*N. gonorrhoeae* cells carrying p*bla*_TEM-1_:


(3) $$\frac{dN\left(t\right)}{dt}=0.018\;N{\left(t\right)}^{1.593}\left(1-\frac{N\left(t\right)}{1100}\right)$$

*N. gonorrhoeae* cells carrying p*bla*_TEM-135_:


(4) $$\frac{dN\left(t\right)}{dt}=0.21\;N{\left(t\right)}^{1.071}\left(1-\frac{N\left(t\right)}{2600}\right)$$

*N. gonorrhoeae* cells carrying p*bla*_TEM-20_ (up to 6 h of growth):


(5) $$\frac{dN\left(t\right)}{dt}=0.012\;N{\left(t\right)}^{1.819}\left(1-\frac{N\left(t\right)}{235}\right)$$

The *R*^2^ values were 0.9916 for wild-type, 0.9958 for p*bla*_TEM-1_, 0.9801 for p*bla*_TEM-135_, and 0.9778 for p*bla*_TEM-20_.

The statistical significance of the difference between the obtained models for describing the data (i.e., the value of *p*) was calculated using the Fisher criterion. The wild-type model outperformed the models for TEM-1, with *p* = 0.02, and TEM-135, with *p* = 0.02. The model for TEM-1 was superior to that of the wild-type and TEM-135 models, with *p* = 0.01. The model for TEM-135 was superior to that of the wild-type model, with *p* = 0.05, and the model for TEM-1, with *p* = 0.06. These results indicate that the developed models adequately describe cell growth for the corresponding strains.

The calculated value of *N_asymp_* differed between wild-type *N. gonorrhoeae* cells and cells carrying the p*bla*_TEM_ plasmids: a maximum *N_asymp_* of 9,300 CFU was observed for wild-type *N. gonorrhoeae*, lower values were observed for *N. gonorrhoeae* carrying the p*bla*_TEM-135_ and p*bla*_TEM-1_ plasmids (2,600 and 1,100 CFU, respectively), and a very low CFU of 235 was observed for *N. gonorrhoeae* carrying the p*bla*_TEM-20_ plasmid. This means that cells carrying p*bla*_TEM_ plasmids had a reduced growth capacity compared to that of cells not carrying these plasmids, which can be explained by the additional energetic and metabolic costs of plasmid reproduction. Moreover, the strain carrying the p*bla*_TEM-20_ plasmid expressing the β-lactamase with the Gly238Ser substitution showed a significantly reduced growth ability compared to that of the wild-type strain and the strains carrying the other plasmids. The reduced viability of the strain carrying the p*bla*_TEM-20_ plasmid was also confirmed by the previously noted characteristics: the cells survived for no more than 6 h when stored on solid medium and did not survive when stored in cryopreservation medium.

### Changes in the Numbers of Viable *Neisseria gonorrhoeae* Cells Carrying Different p*bla*_TEM_ Plasmids in the Presence of Ceftriaxone

**Figure 3:**
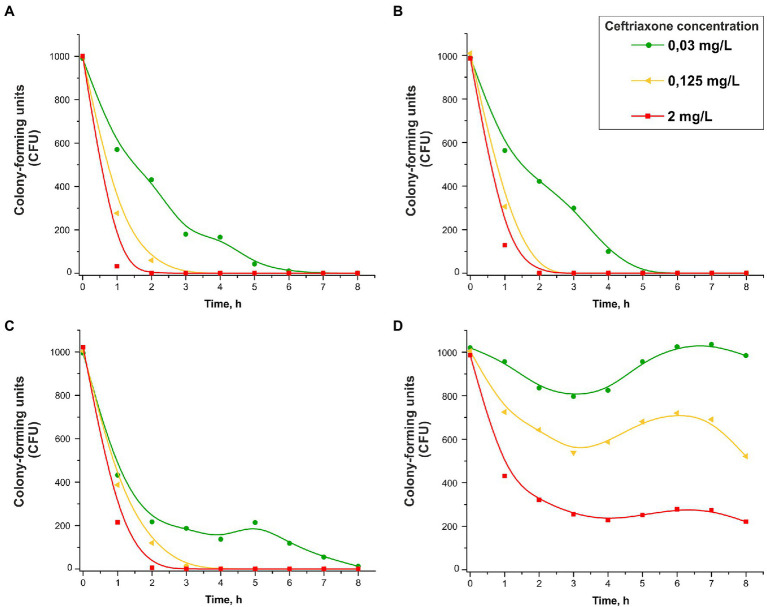
Change in the CFU number vs. time in the presence of 0.03, 0.125, or 2 mg/L ceftriaxone for *Neisseria gonorrhoeae* strain ATCC 49226 not carrying plasmids **(A)**, carrying p*bla*_TEM-1_ **(B)**, carrying p*bla*_TEM-135_ **(C)**, and carrying p*bla*_TEM-20_ (**D**; 37°C, 5% CO_2_). The dots correspond to the mean CFU from three independent experiments (the initial CFU and the standard deviations are provided in Supplementary Table S2).

The changes in the numbers of viable cells over time in the presence of various concentrations of ceftriaxone (0.03, 0.125 and 2 mg/L) are shown in Figure 3. Consistent with the protocol for the bacterial growth studies, the number of CFUs on solid medium (chocolate agar) under various culture conditions, rather than the total number of cells, was determined. As shown in Figure 3A–C, the strains that did not carry ceftriaxone resistance determinants rapidly lost viability in the presence of this antimicrobial agent (the ceftriaxone concentrations used in this experiment were equal to or greater than the MIC_cro_ for these strains, i.e., 0.015–0.03 mg/L; Table 1). After 2–8 h of incubation, the bacterial cells were completely eliminated.

**Scheme 1:**
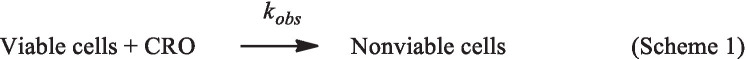
Cell death under the action of ceftriaxone.

Under our experimental conditions, the change in the concentration of ceftriaxone over time can be neglected since this concentration is much higher than that in the cells in the sample: [CRO] = 0.03–2.0 mg/L or (0.045–3.030)·10^−6^ M, *N*_0_ = 1,000 cells (cells at the zero time point). The decrease in CFU over time evidenced in the curves in Figures 3A–C (strains not carrying plasmids and strains carrying p*bla*_TEM-1_ and p*bla*_TEM-135_) followed a logarithmic law, i.e., kinetic curves can be considered applicable for chemical reactions of the first (pseudo-first) order (Scheme 1).

A modified Chick–Watson model developed for disinfection curves (cell death under the action of a disinfecting agent) can be applied to describe the kinetic curves. According to Chick’s law, the dependence of cell survival on time is described by the equations for a first-order chemical reaction [Jensen, 2010; Peleg, 2021; Eqs. (6) and (7)].


(6) $$\frac{dN\left(t\right)}{dt}=-k_{obs}\;N\left(t\right)$$

(7) $$\ln\;\frac{N_{0}}{N}=k_{obs}\;t$$

where *dN*(*t*)*/dt* is the growth rate at a given time, *N*(*t*) is the number of cells at a given time, and *k_obs_* is the observed first-order rate constant.

**Figure 4:**
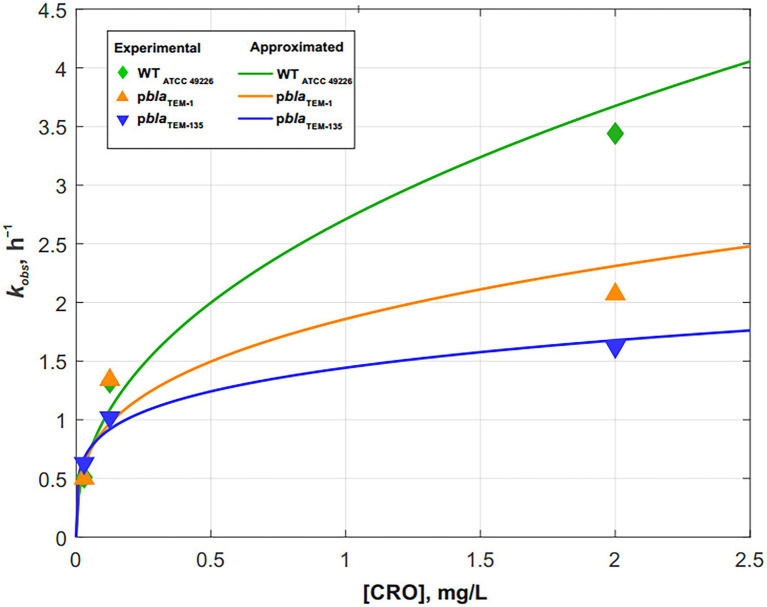
Dependence of the cell death rate constant *k_obs_* on the ceftriaxone concentration for *Neisseria gonorrhoeae* strains not carrying the plasmids and carrying p*bla*_TEM-1_ and p*bla*_TEM-135_. The solid lines show the power approximation curves constructed according to Eqs. (10)–(12).

Fitting the cell death curves (Figures 3A–C) using Eq. (7) made it possible to obtain the values of the observed first-order rate constants $k_{obs}$ for all strains at different concentrations of the disinfecting agent, which in our experiment was ceftriaxone (pseudo-first-order rate constants). The dependence of $k_{obs}$ on the concentration of ceftriaxone is shown in Figure 4.

According to the modified Chick–Watson model, the dependence of the cell death rate on the concentration of ceftriaxone is determined by the following equation:


(8) $$\frac{dN\left(t\right)}{dt}=-k_{d}\;\;{\left[CRO\right]}^{n}N\left(t\right)$$

where [*CRO*] is the ceftriaxone concentration; *n* is the fitting coefficient, also called the coefficient of dilution; and *k_d_* is the true rate constant of cell death.

Thus, the observed death rate constant is related to the true rate constant and the ceftriaxone concentration as follows:


(9) $$k_{obs}=k_{d}\;{\left[CRO\right]}^{n}$$

The dependence of *k_obs_* on the concentration of ceftriaxone, shown in Figure 4, is approximated by the following power functions (*R*^2^–approximation confidence value):

*N. gonorrhoeae* cells not carrying plasmids (wild-type):


(10) $$k_{obs}=2.71\;{\left[CRO\right]}^{0.44}$$

(*R*^2^ = 0.9677)

*N. gonorrhoeae* cells carrying p*bla*_TEM-1_:


(11) $$k_{obs}=1.86\;{\left[CRO\right]}^{0.31}$$

(*R*^2^ = 0.8461)

*N. gonorrhoeae* cells carrying p*bla*_TEM-135_:


(12) $$k_{obs}=1.44\;{\left[CRO\right]}^{0.22}$$

(*R*^2^ = 0.9626)

Thus, the equations describing the decrease in *N. gonorrhoeae* CFU over time under the influence of ceftriaxone are as follows:

*N. gonorrhoeae* cells not carrying plasmids (wild-type):


(13) $$\frac{dN\left(t\right)}{dt}=-2.71\;{\left[CRO\right]}^{0.44}\;N\left(t\right)$$

*N. gonorrhoeae* cells carrying p*bla*_TEM-1_:


(14) $$\frac{dN\left(t\right)}{dt}=-1.86\;{\left[CRO\right]}^{0.31}\;N\left(t\right)$$

*N. gonorrhoeae* cells carrying p*bla*_TEM-135_:


(15) $$\frac{dN\left(t\right)}{dt}=-1.44\;{\left[CRO\right]}^{0.22}\;N\left(t\right)$$

The results showed that the patterns of *N. gonorrhoeae* cell death in the presence of ceftriaxone varied by strain. In the wild-type strain not carrying p*bla*_TEM_, cell death occurred somewhat faster than in the strains carrying p*bla*_TEM-1_ and p*bla*_TEM-135_, although the β-lactamases expressed by these plasmids were not capable of destroying cephalosporins.

However, the CFU reduction vs. time curves for the *N. gonorrhoeae* strain carrying the p*bla*_TEM-20_ plasmid that expresses an ESBL looked completely different (Figure 3D). The MIC of ceftriaxone for this strain was 4 mg/L (Table 1), and at ceftriaxone concentrations of 0.03–0.125 mg/L, the number of viable cells decreased in the first 3 h. Then, a short-term increase in CFU was observed over a period of 3–6 h, and this increase shifted to a reduction in CFU that was similar to the reduction observed in the *N. gonorrhoeae* strain carrying the p*bla*_TEM-20_ plasmid when cultured in the absence of the antibiotic (Figure 2, curve for the strain with the p*bla*_TEM-20_ plasmid). At a ceftriaxone concentration of 0.03 mg/L, i.e., a much lower concentration than the MIC_cro_ for the strain carrying p*bla*_TEM-20_, a significant amount of the antibiotic seemed to be hydrolyzed in the first 3 h, and the number of cells was restored to its initial value during the next 3 h. Moreover, notably, the CFU vs. time curves for this strain did not obey the logarithmic law, and in this case, the Chick–Watson model was not applicable for describing the kinetic curves.

Obviously, the kinetic curves for cells carrying p*bla*_TEM-20_ reflected several processes: hydrolysis of ceftriaxone by the TEM-20 β-lactamase and the growth and gradual death of cells. Therefore, these curves could not be described by a simple Chick–Watson model for cell death in the presence of a disinfecting agent (here, ceftriaxone).

## Discussion

Currently, the threat of the emergence and spread of *N. gonorrhoeae* clinical isolates resistant to third-generation cephalosporins, such as ceftriaxone and cefixime, is a global problem. To date, a few isolates with chromosomal resistance to cephalosporins have been described. The typical ceftriaxone MICs for such isolates are 0.5–1.0 mg/L (Młynarczyk-Bonikowska et al., 2020; Shaskolskiy et al., 2021). However, the emergence of ESBLs that can hydrolyze cephalosporins may lead to a several fold increase in the ceftriaxone MIC.

Although no ESBL-producing *N. gonorrhoeae* isolate has been detected worldwide, the massive use of cephalosporins for gonorrhea treatment in many countries creates the possibility of selecting new variants of TEM β-lactamases. Since the single Gly238Ser mutation could endow blaTEM-135 with the ability to encode an ESBL, such as TEM-20, this allele can be considered a possible precursor of an ESBL. Laboratory evaluation of *N. gonorrhoeae* strains obtained by *in vitro* mutagenesis that produce different types of plasmid β-lactamases, including TEM-20, allows the resistance level to different classes of β-lactam antibiotics to be estimated and the viability of such strains to be assessed. At the same time, such studies carry a high risk of creating a super resistant pathogen in the laboratory conditions. Therefore, special attention should be paid to controlling the safety of research and preventing the spread of the genetically engineered cells into the environment.

In this work, the artificial p*bla*_TEM-1_ and p*bla*_TEM-20_ plasmids were obtained by site-directed mutagenesis of the naturally occurring p*bla*_TEM-135_ Toronto/Rio type plasmid. Efficient plasmid amplification occurred in *E. coli* cells due to the presence of specific replication origins (ori1, ori2, ori3) and the *repA* and *repB* genes encoding replication initiation proteins. The *N. gonorrhoeae* strain ATCC 49226 was transformed with these plasmids using electroporation to obtain individual strains, and the presence of the p*bla*_TEM-1_, p*bla*_TEM-135_, and p*bla*_TEM-20_ plasmids in these strains was confirmed. All manipulations with obtained strains were carried out under strict conditions of biosafety level 3 laboratory. Cells were killed by heat inactivation after experiments.

As expected, benzylpenicillin susceptibility testing showed that compared to the wild-type strain, the strains carrying p*bla*_TEM_ plasmids exhibited high-level resistance (MIC_pen_ ≥ 16 mg/L) to this antibiotic. For all cephalosporins, the MIC values for the strains carrying the p*bla*_TEM-1_ or p*bla*_TEM-135_ plasmid were similar to that for the wild-type strain, i.e., all strains displayed equivalent susceptibility to cephalosporins. However, very different MIC values were obtained for the strain carrying the p*bla*_TEM-20_ plasmid. The MIC of ceftriaxone was 4 mg/L, twice the maximum MIC for the described clinical isolate HO41 with chromosomal ceftriaxone resistance (Ohnishi et al., 2011). The MIC values of the other third- and fourth-generation cephalosporins were as low as 8 mg/L, a pattern that can be explained only by the presence of an enzyme that effectively destroys these antibiotics in cells. Indeed, a single Gly238Ser amino acid substitution in the β-lactamase plasmid gene was sufficient to endow this enzyme with the ability to hydrolyze cephalosporins but retain the ability to hydrolyze penicillins. However, the *N. gonorrhoeae* strain carrying the p*bla*_TEM-20_ plasmid showed substantially reduced viability; cell growth plateaued after 6 h of culture in either the absence or presence of the antibiotic. In addition, the cells did not grow after incubation on plates for more than 6 h or after freezing. Moreover, to determine the MICs, the colonies formed were counted after 24 h of incubation, a possible limitation of this study. The reduced viability of the strain carrying the p*bla*_TEM-20_ plasmid most likely did not affect the measured MICs but decreased the number of proliferated cells.

Our results intersect with the work of Cole et al., 2018, in which genetically engineered *N. gonorrhoeae* strains of the widespread *N. gonorrhoeae* multiantigen sequence typing (NG-MAST) type 1,407, carrying African-type penicillinase-producing plasmids, were obtained. However, the strains described in Cole et al. were unable to retain plasmids in the absence of antibiotic selection, and the cells lost plasmids after even one passage in the absence of penicillin. In our work, the reduced viability of *N. gonorrhoeae* cells carrying the p*bla*_TEM-20_ plasmid was not associated with loss of the plasmid during culture. Moreover, to exclude the effect of cell damage due to the electroporation procedure, all experiments for estimating the viability of the wild-type strain and the strains carrying the p*bla*_TEM-1_, p*bla*_TEM-135_ and p*bla*_TEM-20_ plasmids were performed in triplicate.

The reduced viability of the strain carrying the p*bla*_TEM-20_ plasmid that was demonstrated in this work can be explained by the following reasons: (a) the presence of the plasmid itself, which requires additional reproduction costs in cells and (b) the expression of an ESBL. The action of cephalosporins, like all β-lactam antibiotics, is aimed at inhibiting the synthesis of the bacterial cell wall *via* covalent inhibition of transpeptidase (PBP) activity. The main component of the cell wall is peptidoglycan, which is a macromolecular structure comprising peptide and sugar components. To protect themselves against β-lactam antibiotics, bacteria express β-lactamases, which are localized in the periplasmic space and hydrolyze the C–N bond in the β-lactam ring of the antibiotic, thereby inactivating the drug. Because peptidoglycan in gram-negative bacteria possesses a C-terminal motif in the acyl-D-Ala-D-Ala peptide chain that is structurally analogous to β-lactams, β-lactamases can induce changes in the peptidoglycan composition, thereby reducing the viability of bacterial cells. A change in the structure of the cell membrane leads to the suppression of cell division (bacteriostatic effect) or to the rupture of bacterial cells due to osmotic pressure (bactericidal activity; Sawa et al., 2020). A study published by Fernández et al. (2012) showed that a change in the peptidoglycan structure occurred in *E. coli* cells expressing certain β-lactamase variants: specifically, the level of crosslinked muropeptides was decreased, which negatively affected the viability of these strains.

Evaluation of the resistance of the *N. gonorrhoeae* strains carrying p*bla*_TEM_ plasmids to β-lactam antibiotics also showed that none of the obtained β-lactamase variants, including the ESBL TEM-20, were capable of hydrolyzing carbapenems. Thus, carbapenems remain among the β-lactams that can resist hydrolysis by *N. gonorrhoeae* lactamases, confirming that they are potential drugs for the treatment of gonococcal infections.

The cell growth kinetics were mathematically modeled using the generalized Verhulst equation (Peleg et al., 2007; Peleg and Corradini, 2011). The value of *N_asymp_*, a parameter that characterizes the maximum possible number of cells that can grow under given conditions, was lower for strains carrying p*bla*_TEM_ plasmids than for strains not carrying these plasmids, i.e., the viability of plasmid-carrying strains was reduced compared to that of wild-type *N. gonorrhoeae*. *N_asymp_* decreased in the order of WT—p*bla*_TEM-135_—p*bla*_TEM-1_—p*bla*_TEM-20_. The reduced viability of the strains carrying the p*bla*_TEM_ plasmids may explain why these strains have a lower incidence in the *N. gonorrhoeae* population than strains not carrying these plasmids. The higher viability of *N. gonorrhoeae* carrying p*bla*_TEM-135_ than *N. gonorrhoeae* carrying p*bla*_TEM-1_ may be associated with the presence of the Met182Thr mutation in the β-lactamase, which leads to stabilization of the enzyme structure. As noted previously (Orencia et al., 2001; Cole et al., 2015; Yan et al., 2019), increased enzyme stability may have contributed to the persistence and spread of the TEM-135 β-lactamase variant in the *N. gonorrhoeae* population.

The decrease in the number of viable cells with culture time in the presence of ceftriaxone has been studied. The cell death kinetics of *N. gonorrhoeae* not carrying plasmids and *N. gonorrhoeae* carrying the p*bla*_TEM-1_ and p*bla*_TEM-135_ plasmids, that lack ceftriaxone resistance determinants, can be described by a modified Chick–Watson law for modeling cell death in the presence of a disinfectant (Jensen, 2010; Peleg, 2021). The cell death rate of the wild-type *N. gonorrhoeae* strain in the presence of ceftriaxone was higher than that of the strains carrying the p*bla*_TEM-1_ and p*bla*_TEM-135_ plasmids, although the strains carrying those plasmids were susceptible to ceftriaxone according to the EUCAST criteria.

In contrast, the CFU vs. time curves for the *N. gonorrhoeae* strain carrying the p*bla*_TEM-20_ plasmid that expresses an ESBL cannot be described by a simple Chick–Watson model. The cell death kinetic curves for this strain reflected several processes: hydrolysis of ceftriaxone by the TEM-20 β-lactamase, cell growth, and gradual cell death.

The obtained data on the reduced viability of *N. gonorrhoeae* strains carrying the p*bla*_TEM-20_ plasmid may explain the absence of *N. gonorrhoeae* clinical isolates producing ESBLs that hydrolyze cephalosporins of various generations.

## Data Availability Statement

The original contributions presented in the study are included in the article/Supplementary Material, and further inquiries can be directed to the corresponding author.

## Ethics Statement

Ethical approval/written informed consent was not required for the study of animals/human participants in accordance with the local legislation and institutional requirements.

## Author Contributions

IK performed the experiments, analyzed the results, and wrote the manuscript. DG designed and supervised the project and wrote the manuscript. AV, OA, and JS carried out antimicrobial susceptibility testing. AK and VS supervised work with cell cultures. DD wrote the manuscript. BS directed the project, performed mathematical modeling, and wrote the manuscript. All authors contributed to the article and approved the submitted version.

## Funding

This work was supported by the Russian Science Foundation, grant number 17-75-20039 (plasmid construction, susceptibility testing, mathematical modeling, and cell growth characteristics) and by the Ministry of Science and Higher Education of the Russian Federation to the EIMB Center for Precision Genome Editing and Genetic Technologies for Biomedicine under the Federal Research Program for Genetic Technologies Development for 2019-27, agreement number 075-15-2019-1660 (gene sequencing and analysis of sequence data). Work with cell cultures was performed according to the Ministry of Health of the Russian Federation, assignment number 056-03-2021-124.

## Conflict of Interest

The authors declare that the research was conducted in the absence of any commercial or financial relationships that could be construed as a potential conflict of interest.

## Publisher’s Note

All claims expressed in this article are solely those of the authors and do not necessarily represent those of their affiliated organizations, or those of the publisher, the editors and the reviewers. Any product that may be evaluated in this article, or claim that may be made by its manufacturer, is not guaranteed or endorsed by the publisher.

## Footnotes

^1^ https://www.culturecollections.org.uk/products/bacteria/detail.jsp?refId=NCTC+12700&collection=nctc

^2^ https://www.eucast.org/clinical_breakpoints
